# Evolutionary analyses of visual opsin genes in frogs and toads: Diversity, duplication, and positive selection

**DOI:** 10.1002/ece3.8595

**Published:** 2022-02-07

**Authors:** Ryan K. Schott, Leah Perez, Matthew A. Kwiatkowski, Vance Imhoff, Jennifer M. Gumm

**Affiliations:** ^1^ 7991 Department of Biology York University Toronto Ontario Canada; ^2^ Department of Vertebrate Zoology National Museum of Natural History Smithsonian Institution Washington District of Columbia USA; ^3^ Department of Biology Stephen F. Austin State University Nacogdoches Texas USA; ^4^ 1244 Southern Nevada Fish and Wildlife Office US Fish and Wildlife Service Las Vegas Nevada USA; ^5^ 1244 Ash Meadows Fish Conservation Facility US Fish and Wildlife Service Amargosa Valley Nevada USA

**Keywords:** amphibian, codon‐based likelihood models, photoreceptor, sensory biology, spectral tuning, visual pigments

## Abstract

Among major vertebrate groups, anurans (frogs and toads) are understudied with regard to their visual systems, and little is known about variation among species that differ in ecology. We sampled North American anurans representing diverse evolutionary and life histories that likely possess visual systems adapted to meet different ecological needs. Using standard molecular techniques, visual opsin genes, which encode the protein component of visual pigments, were obtained from anuran retinas. Additionally, we extracted the visual opsins from publicly available genome and transcriptome assemblies, further increasing the phylogenetic and ecological diversity of our dataset to 33 species in total. We found that anurans consistently express four visual opsin genes (*RH1*, *LWS*, *SWS1*, and *SWS2*, but not *RH2*) even though reported photoreceptor complements vary widely among species. The proteins encoded by these genes showed considerable sequence variation among species, including at sites known to shift the spectral sensitivity of visual pigments in other vertebrates and had conserved substitutions that may be related to dim‐light adaptation. Using molecular evolutionary analyses of selection (d_N_/d_S_) we found significant evidence for positive selection at a subset of sites in the dim‐light rod opsin gene *RH1* and the long wavelength sensitive cone opsin *LWS*. The function of sites inferred to be under positive selection are largely unknown, but a few are likely to affect spectral sensitivity and other visual pigment functions based on proximity to previously identified sites in other vertebrates. We also found the first evidence of visual opsin duplication in an amphibian with the duplication of the *LWS* gene in the African bullfrog, which had distinct *LWS* copies on the sex chromosomes suggesting the possibility of sex‐specific visual adaptation. Taken together, our results indicate that ecological factors, such as habitat and life history, as well as behavior, may be driving changes to anuran visual systems.

## Graphical abstract

Frogs and toads are highly visual animals, but the diversity, function, and evolution of their visual systems are understudied compared to other vertebrates. We used field collected samples and existing genomic resources to investigate the molecular evolution and diversity of visual opsin genes across frogs and toads. We found considerable variation and evidence for potential functional adaptation to different light environments and behaviors, laying the groundwork for future studies of visual ecology and evolution in this group.

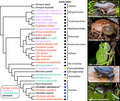

## 1 INTRODUCTION

Frogs and toads (Amphibia: Anura) were used as early model systems for studies of the vertebrate visual system, and many core mechanisms of visual function in vertebrates were discovered using anuran models, yet they have largely fallen out of use in vision biology (for a review see Donner & Yovanovich, 2020). Relatively few modern studies have examined anuran visual systems despite the importance of vision to many aspects of anuran biology, including movement patterns, habitat preferences, foraging, reproduction, and possibly thermoregulation (Buchanan, 2006). Anurans also have broad phenotypic, ecological, and behavioral diversity (Anderson & Wiens, 2017; Hödl & Amézquita, 2001; Moen, 2019), which suggests that their visual systems may have adapted to contend with different light environments and functional demands. Several recent studies have investigated evolutionary correlations between species ecology and both morphological (eye size; Huang et al., 2019; Shrimpton et al., 2021; Thomas et al., 2020) and spectral (lens transmission and pigmentation; Yovanovich et al., 2020; Thomas et al., 2022) features of anuran eyes. These studies found significant variation in anuran eye size and lens transmission that are associated with differences in behavior and ecology suggesting substantial adaptation in visual function among anuran lineages. However, the molecular mechanisms underlying morphological and spectral adaptation in anuran visual systems have not yet been explored using a comparative evolutionary approach.

Here we focus on the molecular evolution of the visual opsin genes. These genes encode the protein component of visual pigments, the molecules contained in the photoreceptor cells of the retina that absorb light and initiate the phototransduction cascade that results in vision. In vertebrates there are ancestrally five visual opsin genes: one expressed in the dim‐light sensitive, rod photoreceptors (*RH1*), and four expressed in spectrally distinct bright‐light sensitive, cone photoreceptors (*LWS*, *RH2*, *SWS1*, *SWS2*). The different visual pigments formed by each of these opsins absorb light maximally (*λ*_max_) at different wavelengths, and these differences are controlled by the structure of the opsin protein as well as by the nonprotein component of the visual pigment, the light‐sensitive chromophore (Bowmaker, 2008). Visual opsins have been independently lost and duplicated in many different vertebrate lineages, resulting in as few as one visual opsin gene in some lineages, such as deep diving whales (Meredith et al., 2013), and up to 38 *RH1* copies in the spinyfin, *Diretmus argenteus* (Musilova et al., 2019). Further, variation in the sequences of opsin genes among species can result in considerable differences in *λ*_max_ among species. This variation in the number and type of visual opsins is one of the primary ways vertebrates can adapt their visual systems to different spectral environments (Bowmaker et al., 1994; Loew et al., 2002; Loew & Lythgoe, 1978).

Shifts in spectral sensitivity of a particular visual opsin are termed spectral tuning and have been identified in all major vertebrate lineages (Davies et al., 2012; Yokoyama, 2008). Spectral tuning can occur via changes to the opsin‐coding sequence that result in the substitution of amino acid residues, particularly those lining the chromophore‐binding pocket formed by the opsin's seven transmembrane α‐helices, and alter the interaction between the opsin and the chromophore. Shifts in the spectral sensitivity of visual pigments can play an important role in the evolution, ecology, and behavior of species. The most extreme example is in African lake cichlids where evidence suggests that divergent selection on spectral sensitivity in LWS drove speciation of two Lake Victoria cichlids through sensory drive (Seehausen et al., 2008). In neotropical cichlids, visual pigments have also been shown to be under divergent selective pressures associated with differences in habitat and light environments (Escobar‐Camacho et al., 2017; Hauser et al., 2017, 2021; Schott et al., 2014; Torres‐Dowdall et al., 2015). In other vertebrates, similar associations between positive and divergent selection on opsin genes and shifts in light environments and behaviors have been found in diverse groups including in snakes (Schott et al., 2018), geckos (Schott et al., 2019), bats (Gutierrez, Castiglione, et al., 2018; Gutierrez, Schott, et al., 2018), whales (Dungan et al., 2016; McGowen et al., 2020), warblers (Bloch et al., 2015), and many other examples in teleost fishes (reviewed in Carleton et al., 2020).

In addition to spectral tuning, changes to the opsin sequence can also affect other aspects of visual pigment function including kinetic rates, such as light and thermal activation. For example, in the rod opsin (RH1) a D83N substitution has been identified as a potential dim‐light adaptation by accelerating the formation of the active, signaling state of the visual pigment upon light activation (Sugawara et al., 2010). The effect of this mutation has been explored in a number of different groups that inhabit dim‐light environments including cichlid fishes, bats, whales, echidnas, and bowerbirds (Bickelmann et al., 2012; Dungan & Chang, 2017; Hauser et al., 2017; Sugawara et al., 2010; van Hazel et al., 2016). Like spectral tuning, these other functional properties of visual pigments may play an important role in visual adaptation but have been comparatively understudied.

Relative to other vertebrates, little is known about the diversity of photoreceptors and visual opsins in anurans and other amphibians. Four of the five visual opsin genes have been identified in anurans (*RH1*, *LWS*, *SWS1*, *SWS2*), but *RH2* has not been found in any amphibian and is presumed to have been lost early during their evolution (Bowmaker, 2008; Schott et al., 2021). These opsins may be found in as many as eight different photoreceptor types including two types of rods, one of which is unique to amphibians. The typical, RH1, rods (also called red rods) contain a green‐absorbing pigment (*λ*_max_ of 491–503 nm; Table 1; Liebman & Entine, 1968; Siddiqi et al., 2004) that is formed from rod opsin (RH1). The second, novel, type of rod, historically (and confusingly) called a green rod, contains a blue‐absorbing visual pigment (*λ*_max_ of 430–440 nm; Muntz & Reuter, 1966; Dartnall, 1967; Liebman & Entine, 1968; Hisatomi et al., 1999; Darden et al., 2003; Govardovskii & Reuter, 2014) that is formed from the SWS2 opsin typically expressed in cone photoreceptors. These SWS2 rods are rarer than the RH1 rods, but their proportion of the total rod population is highly variable in the limited number of species that have been studied to date (3–20%; Denton & Wyllie, 1955; Nilsson, 1964; Röhlich & Szél, 2000), and this rod type may not be present in all frogs (e.g., *Oophaga pumilio*; Siddiqi et al., 2004). Further, the SWS2 opsin of at least some frogs, but none of the salamanders examined so far, have a unique amino acid residue, Thr47, that results in highly reduced thermal activation rates close to the level of RH1 opsins and much lower than any other cone opsins (Kojima et al., 2017).

**TABLE 1:** Maximum spectral sensitivity (*λ*_max_ in nm) of adult anuran photoreceptors estimated through microspectrophotometric (MSP) or electroretinographic (ERG) methodologies. Photoreceptors are grouped into rods and cones and then further divided based on *λ*_max_

Species	Rod 1	Rod 2	Cone 1	Cone 2	Cone 3	Reference
*Bufo bufo*	502	432				Govardovskii et al. (2000)
*Hyla cinerea*	503	435				King et al. (1993)
*Lithobates catesbeianus*	502	432	570		433	Govardovskii et al. (2000); Hárosi (1982)
*L. pipiens*	502–503	432	575	~500		Govardovskii et al. (2000); Liebman and Entine (1968)
*L. ridibunda*	502	433				Govardovskii et al. (2000)
*L. sphenocephalus*	501, 505	~437	579, 603			Schott et al. (2021)
*Rana temporaria*	501–503	434	562		431	Govardovskii et al. (2000); Koskelainen et al. (1994)
*Oophaga pumilio*	491		561	489	466	Siddiqi et al. (2004)
*Rhinella marinus*	503	432				Govardovskii et al. (2000)
*Xenopus laevis*	523–524 (A_2_)	444–445 (A_2_)	611 (A_2_)			Govardovskii et al. (2000); Witkovsky et al. (1981)

Frogs also have at least three, and up to six, types of cones that include up to four different visual pigments. This includes red‐sensitive LWS pigments (*λ*_max_ of ~560–575 nm; Liebman & Entine, 1968; Liebman, 1972), a green absorbing pigment spectroscopically indistinguishable from that in the RH1 rods (*λ*_max_ of ~500 nm), and a blue‐absorbing pigment with a *λ*_max_ of ~430 nm (Hárosi, 1982; Koskelainen et al., 1994; Liebman & Entine, 1968). While the opsin identities of the visual pigments contained in all of the cones have not been determined, it seems highly likely that the green‐sensitive cones contain the RH1 opsin also present in the RH1 rods, making this a rare example of the RH1 pigment being contained in a cone photoreceptor (de Busserolles et al., 2017; Schott et al., 2016). The blue cones could contain either SWS1 or SWS2 visual pigments, and it is possible that both types of cones are present, at least in some species. SWS1 expression has been detected in cones in both *Xenopus laevis* and in bullfrog (*Lithobates catesbeianus*; Hisatomi et al., 1998; Starace & Knox, 1998). Direct evidence of SWS2 cones has not been found in frogs but has been detected in salamanders (Isayama et al., 2014). Spectroscopically, three types of cones were identified in *Oophaga pumilio* (Siddiqi et al., 2004) that had *λ*_max_ of ~561, ~489, and ~466 nm. Only LWS is known to absorb maximally longer wavelengths (e.g., >550 nm), but the identities of the visual pigments in the 489 and 466 nm cones are less clear and could be some combination of RH1, SWS2, or SWS1.

To date, the photoreceptor and visual pigment complements of frogs have yet to be adequately resolved and almost no data are available on variation among species. Here we sequence visual opsins from 14 North American anuran species representing six families. We also take advantage of growing anuran genomic and transcriptomic resources to extract visual opsins from 14 species, which when combined with sequences available on Genbank, resulted in a total sample from 33 species and 12 families (out of 55 currently recognized families). While this is still a small portion of total anuran diversity, our study species represent diverse evolutionary lineages and life histories, and thus we hypothesize they possess visual systems adapted to meet different ecological needs. We aim to: (1) determine which opsin genes are expressed in anuran retinas; (2) identify variation in opsin sequences among anuran species, including at potential spectral tuning and other functionally relevant sites; and (3) test for evidence of positive selection that may indicate functional adaptation to the distinct light environments inhabited by our study species.

## 2 METHODS

### 2.1 Sample collection

**FIGURE 1:**
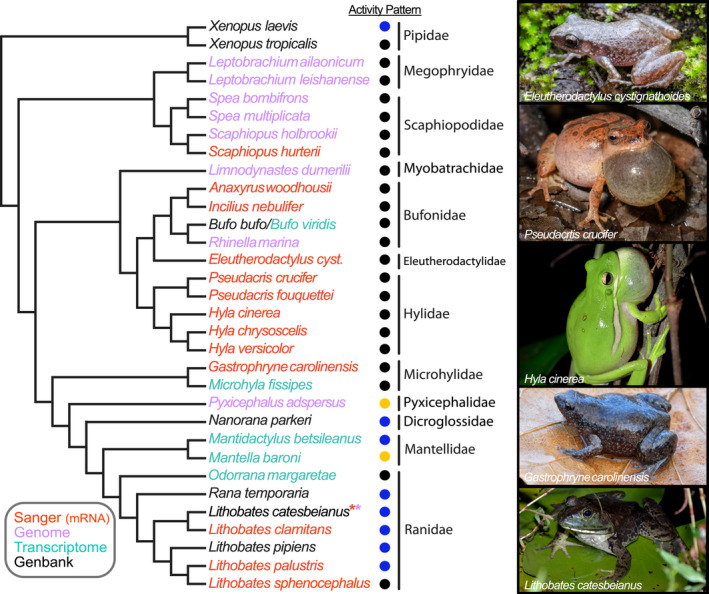
Phylogenetic tree illustrating evolutionary relationships among the study species based upon several recent large‐scale phylogenetic studies (Feng et al., 2017; Jetz & Pyron, 2018; Pyron & Wiens, 2011; Streicher et al., 2018). The activity pattern of species is denoted with a circle where black = primarily nocturnal, yellow = primarily diurnal, and blue = both. The source of the sequence is also indicated through the color of the species names (the asterisks indicate that *L*. *catesbeianus* data were obtained from multiple sources). Sanger sequences were newly sequenced for the present study, while those from genomes and transcriptomes were newly extracted from existing assemblies. Sequences obtained from Genbank may have ultimately been derived from Sanger or whole genome sequencing. Photographs by MAK

Thirteen of the 14 anuran species newly sampled in this study are native to eastern Texas where they were collected. These include two species of “true toad” (*Incilius nebulifer* and *Anaxyrus woodhousii*); two species of chorus frog (*Pseudacris crucifer* and *P*. *fouquettei*); three species of treefrog (*Hyla chrysoscelis*, *H*. *versicolor*, and *H*. *cinerea*); four species of pond frog (*Lithobates catesbeianus*, *L*. *clamitans*, *L*. *palustris*, *and L*. *sphenocephalus*); one species of narrowmouth toad (*Gastrophryne carolinensis*); and one species of spadefoot toad (*Scaphiopus hurterii*). In addition to the 13 native eastern Texas species, this study also includes the chirping frog *Eleutherodactylus cystignathoides*, which is introduced in eastern Texas, but native to the Rio Grande Valley in southern Texas. Our sampling also includes species for which genomic and transcriptomic resources are publicly available (see below). The phylogenetic relationships among study species are depicted in Figure 1.

For the Texas frogs, up to five individuals per species were collected throughout the study period, from autumn of 2017 through spring of 2019. Most individuals were collected from ephemeral breeding ponds in the Stephen F. Austin Experimental Forest, which is part of the Angelina National Forest, and the adjacent Alazan Bayou Wildlife Management Area in southwestern Nacogdoches County, TX, USA. The strictly urban *E*. *cystignathoides* were collected on or near the Stephen F. Austin State University campus. All study animals were collected under permit and in compliance with the U.S. Forest Service, Texas Parks and Wildlife Department, and Nacogdoches city law enforcement. Following protocols described by the Herpetological Animal Care and Use Committee (2004) of the American Society of Ichthyologists and Herpetologists (ASIH), and approved by the SFASU Institutional Animal Care and Use Committee (Protocol # 2017‐007), animals were euthanized via overdose of the anesthetic Tricaine methanesulfonate (MS‐222). Euthanasia was confirmed prior to eye dissection by severing and pithing the spinal cord. Upon removal from the eye, each retina was immediately stored at −20°C in RNAlater (Thermo Fisher Scientific, Waltham, MA, USA).

### 2.2 Opsin sequencing

Total retinal mRNA was extracted from one of each study animal's retinas with an RNeasy Mini Kit and QIAshredder (Qiagen, Valencia, CA, USA), quantified with a NanoVue spectrophotometer (GE Healthcare, Chicago, IL, USA), and stored at −80°C; the second retina remained in storage in RNAlater at −20°C. Aliquots containing 0.4 μg mRNA were reverse transcribed using SuperScript™ IV Reverse Transcriptase (Invitrogen, Carlsbad, CA, USA) with an oligo(dT) primer to synthesize 20 μl aliquots of total cDNA. Fragments of each opsin‐coding gene were amplified via polymerase chain reactions (PCR) for sequencing. Gene‐specific and degenerate primers for anuran *RH1*, *LWS*, *SWS1*, and *SWS2* (Schott et al., 2022a) were designed using Primer3 (Rozen & Skaletsky, 1999) from aligned GenBank reference sequences.

Target gene fragments were amplified in a Mastercycler ep realplex thermocycler (Eppendorf, Hamburg, Germany). PCR products were purified with the Wizard^®^ SV Gel and PCR Clean‐Up System (Promega Corporation, Madison, WI, USA), quantified, and prepared according to specifications set by the DNA Sequencing Facility at the University of Texas at Austin for Sanger sequencing (Sanger et al., 1977). Returned partial sequences were identified to the gene via nucleotide BLAST (Altschul et al., 1990). In the case of *Lithobates clamitans*, only one of the two individuals collected was sequenced. Among other species, opsins were sequenced from two individuals in *Incilius nebulifer*, *Eleutherodactylus cystignathoides*, *Hyla chrysoscelis*, *H*. *versicolor*, *Gastrophryne carolinensis*, and *L*. *clamitans*; three individuals in *Anaxyrus woodhousii*, *H*. *cinerea*, *Pseudacris fouquettei*, *L*. *sphenocephalus*, and *L*. *palustris*; and four individuals in *P*. *crucifer* and *Scaphiopus hurterii*. Prior to further analysis, partial sequences of the same gene from the same species were cleaned and merged into a consensus sequence in Geneious 10 (Biomatters, Ltd., Auckland, New Zealand; Kearse et al., 2012). Complete lab protocols used for opsin sequencing are available on protocols.io (Schott et al., 2022b).

### 2.3 Visual opsin gene datasets

Additional visual opsin sequences were obtained from the NCBI Genbank database and were extracted from all available anuran genome and transcriptome assemblies using BLAST (Table 2, Schott et al., 2022a). We also assembled *Mantidactylus betsileanus* transcriptome reads (from Wollenberg Valero et al., 2017) *de novo* using Trinity v2.8.5 (Grabherr et al., 2011) and extracted visual opsin coding regions from the resulting assembly. Total number of sequences obtained for each opsin, and their source, can be found in the Zenodo dataset (Schott et al., 2022a).

**TABLE 2:** Summary of visual opsin genes sequenced or extracted in the current study. Full details, including individual accession numbers can be found on Zenodo (Schott et al., 2022a)

Species	RH1	LWS	SWS2	SWS1	Sequence source	Reference
*Anaxyrus woodhousii*	●		●	●	mRNA	This study
*Bufo bufo*	●				mRNA	Genbank
*Bufo viridis*	^a^	●	●	●	Transcriptome	Gerchen et al. (2016)
*Eleutherodactylus cystignathoides*	●	●	●	●	mRNA	This study
*Gastrophryne carolinensis*	●	●	●	●	mRNA	This study
*Hyla chrysoscelis*	●	●	●	●	mRNA	This study
*Hyla cinerea*	●	●	●	●	mRNA	This study
*Hyla versicolor*	●	●	●	●	mRNA	This study
*Incilius nebulifer*	●	●	●	●	mRNA	This study
*Leptobrachium ailaonicum*	●	●	●	●	Genome	Li, Ren, et al. (2019)
*Leptobrachium leishanense*	●	●	●	●	Genome	Li, Yu, et al. (2019)
*Limnodynastes dumerilii*	●	●	●	●	Genome	Li et al. (2020)
*Lithobates catesbeianus*	●	●	●	●	mRNA, Genome	Kayada et al. (1995); Hisatomi et al. (1998); Hisatomi et al. (1999); Hammond et al. (2017)
*Lithobates clamitans*	●	●	●	●	mRNA	This study
*Lithobates palustris*	●	●	●	●	mRNA	This study
*Lithobates pipiens*	●				mRNA	Pittler et al. (1992)
*Lithobates sphenocephalus*	●	●	●	●	Transcriptome	Schott et al. (2021)
*Rana temporaria*	●				mRNA	Genbank
*Mantella baroni*			●		mRNA	Kojima et al. (2017)
*Mantidactylus betsileanus*	●	●	●		Transcriptome	Wollenberg Valero et al. (2017)
*Microhyla fissipes*	●	●	●	●	Transcriptome	Zhao et al. (2016)
*Nanorana parkeri*	●	●	●	●	Genome	Genbank
*Odorrana margaretae*	●	●	●	●	Transcriptome	Qiao et al. (2013)
*Pseudacris crucifer*	●	●	●	●	mRNA	This study
*Pseudacris fouquettei*	●		●	●	mRNA	This study
*Pyxicephalus adspersus*	●	●	●	●	Genome	Denton et al. (2018)
*Rhinella marina*	●	●	●	●	mRNA, Genome	Edwards et al. (2018)
*Scaphiopus holbrookii*	●	●	●	●	Genome	Seidl et al. (2019)
*Scaphiopus hurterii*	●	●			mRNA	This study
*Spea bombifrons*	●	●	●	●	Genome	Seidl et al. (2019)
*Spea multiplicata*	●	●	●	●	Genome	Seidl et al. (2019)
*Xenopus laevis*	●	●	●	●	mRNA, Genome	Session et al. (2016)
*Xenopus tropicalis*	●	●	●	●	Genome	Hellsten et al. (2010)

^a^ Partial sequence was recovered but not used in analyses.

For selection analyses in PAML, we generated gene trees for each opsin (“gene tree”) and generated topologies for each gene that reflect the current understanding of species relationships depicted in Figure 1 (“evolutionary tree”). Because individual gene trees do not always reflect species’ evolutionary histories, it is a common approach in selection analyses to compare results from both types of topologies to ensure results are robust to minor topological differences (Schott et al., 2018, 2019). Coding regions for each of the four visual opsin genes obtained from anurans (*RH1*, *LWS*, *SWS1*, *SWS2*) were aligned using MUSCLE codon alignment as implemented in MEGA (Edgar, 2004; Tamura et al., 2011) followed by manual correction. Maximum likelihood (ML) gene trees were inferred for each gene using PhyML 3 (Guindon et al., 2010) under the GTR + G + I model with a BioNJ starting tree, the best of NNI and SPR tree improvement, and aLRT SH‐like branch support (Anisimova & Gascuel, 2006). For the evolutionary tree, we generated a topology for each gene that matched the expected species relationships based on the large‐scale phylogenies of Pyron and Wiens (2011), Feng et al. (2017), Jetz and Pyron (2018), and Streicher et al. (2018).

### 2.4 Selection analyses

To estimate the strength and form of selection acting on the visual opsin genes in anurans, each dataset was analyzed using codon‐based likelihood models from the codeml program of the PAML 4 software package (Yang, 2007). Specifically, we used the random sites models (M0, M1a, M2a, M2a_rel, M3, M7, M8a, and M8) to infer alignment‐wide selection patterns and to test for positive selection acting on any of the genes. All analyses were run with varying starting values to avoid potential local optima. To determine significance, model pairs were compared using a likelihood ratio test (LRT) with a *χ* ^2^ distribution. To ensure results were robust to minor differences in tree topology, analyses were run using both the ML gene trees and the evolutionary tree topologies for each opsin, modified to contain the basal trichotomy required by PAML. The Bayes Empirical Bayes (BEB) approach was used to identify individual sites with a high posterior probability of being in the positively selected class of sites.

We also analyzed the data using the HYPHY model FUBAR (Murrell et al., 2013; Pond & Frost, 2005) implemented on the Datamonkey webserver (Delport et al., 2010). This model uses a hierarchical Bayesian method to average over a much larger number of site classes than the PAML models and importantly allows for an independently estimated value for *d*_S_. The FUBAR selection analyses generated a gene tree inferred under the GTR model.

## 3 RESULTS

### 3.1 Frog visual opsins

Partial coding sequences of four opsins—RH1, LWS, SWS1, and SWS2—were recovered from the retinal mRNA of 14 anuran species (Table 2, Schott et al., 2022a). Several primer pairs were unsuccessful, and we failed to amplify sequences, or parts of sequences, from a number of species (Table 2, Schott et al., 2022a). Consequently, we do not consider the lack of recovery of any of the opsins genes from retinal mRNA as evidence for a lack of expression or gene loss. Additional coding sequences were extracted from available frog genomes and transcriptomes, as well from Genbank. This resulted in 31 RH1, 28 LWS, 26 SWS1, and 30 SWS2 sequences in total (Table 2, Schott et al., 2022a).

**FIGURE 2:**
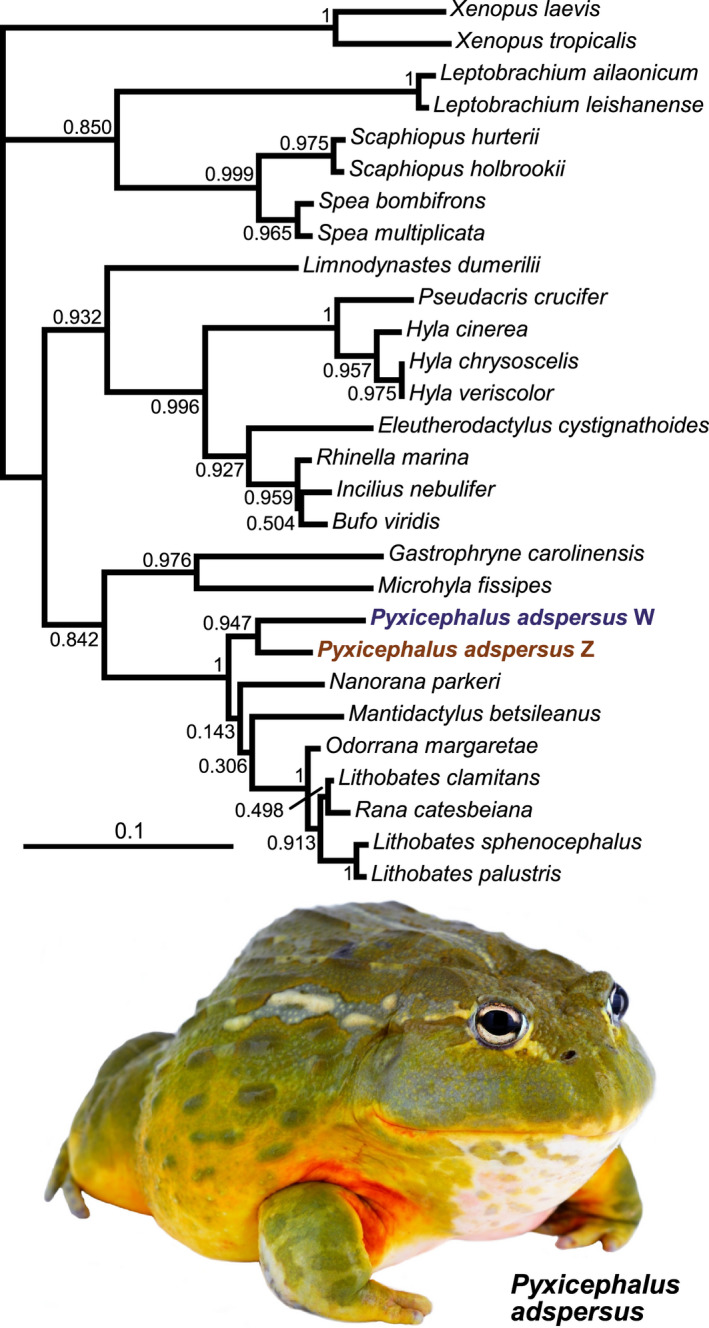
Maximum likelihood gene tree for *LWS* depicting the two *LWS* genes in *Pyxicephalus adspersus*. The gene tree was inferred using PhyML 3 (Guindon et al., 2010) under the GTR + G + I model with a BioNJ starting tree, the best of NNI and SPR tree improvement. Branch support values (aLRT SH‐like; Anisimova & Gascuel, 2006) are shown at the nodes. The basal trichotomy is required by PAML and was manually created. Photograph by John Clare

For each of the available frog genomes all four expected visual opsins (*RH1*, *LWS*, *SWS1*, and *SWS2*) were recovered. In the *Pyxicephalus adspersus* genome we identified two *LWS* genes, one on each of the two sex chromosomes (W and Z). The two sequences are relatively divergent sharing 93.5% amino acid identity (91.8% nucleotide identity), and the Z chromosome sequence has a single amino acid deletion at site 331 (note, site numbering is relative to bovine rhodopsin throughout). Phylogenetic analyses revealed the sequences are most closely related to each other suggesting that they are a species‐specific (or at least lineage‐specific) duplication (Figure 2).

### 3.2 Variation at known spectral tuning sites

Each of the four visual opsins possessed at least one amino acid substitution at a gene‐specific site known in other vertebrates to tune spectral sensitivity of visual pigments (Table 3). The RH1 gene exhibited a change from the nonpolar, aliphatic amino acid alanine (A) to the polar, uncharged serine (S) at position 299 (notated as A299S) in eight species (Table 3, Schott et al., 2022a). This change is responsible for a slight (2 nm) shift in bovine and cetacean RH1 (Dungan & Chang, 2017) and has been implicated in spectral tuning in deep dwelling teleost fishes (Hunt et al., 1996, 2001). The substitution Y102F was found in both *Leptobrachium* species. This change may produce a slight blue‐shift, perhaps in combination with another change not found in frogs (Y96V; Yokoyama, 2008). The substitution L194P occurs in *Microhyla fissipes*. This site has been identified as a spectral tuning site in RH1, but the documented substitution is P194R, and it may only have an effect in combination with other residues (Yokoyama, 2008). Additionally, anuran RH1 varied at six amino acid positions (46, 52, 93, 97, 109, 116) known to affect the spectral sensitivity of other vertebrate visual pigments (Table 3).

**TABLE 3:** Variation in anuran opsin sequences at known spectral tuning sites (based on those identified in Yokoyama, 2008). The residues we identified in anurans are listed for each spectral tuning site, while those sites with variation in the same opsin are bolded. Site numbers are based on bovine RH1 numbering

Site (RH1 numbering)	Known from	Known spectral variants	Residues in anurans			
			RH1	SWS1	SWS2	LWS
44	SWS2	M/T	M	M	M	M
46	SWS1/2	F/T/L	L/M	**V/M/A/F**	F	F
49	RH2, SWS1	S/F/A/V/L	L	**L/I/F/V**	I	A/I/G/L/F/S
52	RH2, SWS1	L/M/T/F	F/L	**T/A**	F	V/C/I
83	RH1/2	D/N	N	G	N	D
86	RH2, SWS1	M/T/F/S/L/Y	M	**M/I**	V	E
90	SWS1	S/C	G	S	G	A
91	SWS1/2	V/I/S/P	F	**I/N**	S	S
93	SWS1	T/P/L/I	I/V	**T/I/V/P**	T/V/M	I
94	SWS2	A/S/C	T	V	A	S
96	RH1	Y/V	Y	V/I/M	Y	F/I/A/V/C
97	RH2, SWS2	T/A/S/C	T/S	S/N	S	N
102	RH1	Y/F	**Y/F**	Y/C	Y	Y
109	SWS1/2	V/A/G	G/T	**V/A/F/T**	A	L/M
113	SWS1	E/D	E	**E**	E	E
114	SWS1	A/G	G	**G/A**	G	G
116	SWS1/2	L/V/T	F/C	**V/I/T**	T	T
118	SWS1/2	S/T/A/G	T	**T/S**	T	S/A
122	RH1, SWS1/2	E/I/Q/M	E	**L**	**M/I**	I
124	RH1	A/S/G/V	A	T/I	S/G	G/A
132	RH1	A/S	A	A	A	A
164	RH2, LWS	S/A	A	G	G/S/A	**A/S**
181	LWS	H/Y	E	E	E	H
194	RH1	P/R	**L/P**	V/I	V	G
195	RH1	N/A	K	G	N	S/N
207	RH2	M/L	M	I/V	M/I/L	L
208	RH1	F/Y	F	F	F	M
211	RH1	H/C	H	C	C	C
261	RH1, SWS2, LWS	F/Y	F	F	F	Y
265	SWS2	W/Y	W	Y	W	W
269	SWS2, LWS	A/S/T	A	A	A	T
292	RH1/2, SWS2, LWS	A/S	A	A	S	A
295	RH1	A/S	A	S	S	A
299	RH1	A/S	**A/S**	C	T	T
300	RH1	I/T/L	I	V	V	I

On the LWS opsin, an amino acid change occurred at known LWS tuning site 164 (anuran LWS‐specific site 179), at which position 13 species expressed A, while remaining species expressed S (Table 3, Schott et al., 2022a). The substitutions A164S and S164A were shown to shift *λ*_max_ by 6 and −7 nm, respectively, in mammalian LWS (Asenjo et al., 1994; Yokoyama, 2008; Yokoyama et al., 2005). Anuran LWS also varied at three RH1 tuning sites (96, 124, and 195), two RH2/SWS1 tuning sites (49 and 52), and two SWS1/2 tuning sites (109 and 118), many of which include non‐conservative amino acid substitutions and known spectral variants that could be expected to effect *λ*_max_ (Table 3).

SWS1 exhibited the greatest number of amino acid changes at gene‐specific tuning sites (Table 3, Schott et al., 2022a). All 10 variable SWS1‐specific sites (46, 49, 52, 86, 91, 93, 109, 114, 116, and 118) occurred within the first three transmembranes. At site 46 (anuran SWS1‐specific site 42), the species expressed one of four residues, although none of these includes the known SWS1 spectral variant (F46T; Table 3; Yokoyama, 2008). Site 49, which varied among four residues in our sample (L, I, F, V), did include the known spectral variants F49V/L (Table 3). The substitutions F49V (in birds) and F49L (in mammals) are responsible for a shift from ultraviolet *λ*_max_ (~360 nm) to violet *λ*_max_ (390+ nm) in combination with substitutions at several other sites (Yokoyama, 2008). Sites 52, 86, and 91 were less variable and did not have known variants (Table 3). There were four residues found at site 93 (T, I, V, P) that include known spectral variants T93P and I93T (Table 3). Only four species had P93, three had I, five have V, and the rest T (Table 3; Schott et al., 2022a). The T93P substitution was shown to contribute to the red‐shifted *λ*_max_ of *X*. *laevis* SWS1 but may have little effect in isolation (Takahashi & Yokoyama, 2005). The substitution I93T was shown to cause a −6 nm shift in elephant SWS1 (Yokoyama et al., 2005). The effects of the other residues found in anurans at this site are not known. Site 109 had four variants in anurans (V, A, F, T). The substitution V109A was also identified as contributing to the violet *λ*_max_ of *X*. *laevis* SWS1, but similarly in isolation had no effect (Yokoyama et al., 2005). At site 114 two variants were found (A and G), and the substitution A114G was shown to result in a 5 nm shift in an inferred ancestral SWS1 pigment (Shi & Yokoyama, 2003). Sites 116 and 118 varied at three (V, I, T) and two (T, S) sites, respectively, and substitutions at both sites contribute to the red‐shifted *λ*_max_ of *X*. *laevis* SWS1 in coordination with substitutions at other sites but were not found to have individual effects (Takahashi & Yokoyama, 2005). Finally, in addition to variation at the aforementioned tuning sites, anuran SWS1 also varied at known RH1 tuning sites 96, 102, 124, 194, RH2 site 207, and RH2/SWS2 tuning site 97 (Table 3).

On the SWS2 opsin, amino acid variation occurred at gene‐specific tuning site 122 (anuran SWS2‐specific site 131), with 10 species expressing I and remaining species expressing M (Table 3, Schott et al., 2022a). The substitution I122M resulted in a −6 nm shift in newt (*Cynops pyrrhogaster*) SWS2 (Takahashi & Ebrey, 2003). In addition, anuran SWS2 varied at four amino acid positions (93, 124, 164, and 207) known in other opsins to affect spectral sensitivity (Table 3).

### 3.3 Variation at other functionally relevant sites

RH1 site 83 has been suggested to be associated with dim‐light adaptation through a D83N substitution (Sugawara et al., 2010). The anurans we sampled all had N83. S299A (and vice versa) was found to affect retinal release rate in mammals (Dungan & Chang, 2017) and the sampled anurans varied among these two residues. Additionally, all frogs in our sample have SWS2 with T47, a mutation that was shown to result in increased dark state stability (low thermal isomerization rate; Kojima et al., 2017). Other sites known to affect kinetic rates, such as RH1 sites 59, 288, and 292 (Castiglione et al., 2017; Dungan & Chang, 2017) were conserved in our sample of frogs.

In all four opsins, amino acid changes also occurred at additional sites forming the chromophore‐binding pocket, and thus these substitutions are likely to affect visual pigment function (list of sites provided in Hunt et al., 2001). These included two sites (54 and 119) on RH1, two sites (119 and 160) on LWS, six sites (47, 82, 120, 258, 271, and 307) on SWS1, and two sites (207 and 258) on SWS2. Variation at site 119 included polarity changes in both RH1, with the amino acid change L119V in *G*. *carolinensis* and *M*. *fissipes*, and LWS, with the change V119T in several species (Table 3, Schott et al., 2022a). Another polarity change occurred at LWS site 160, at which few species have a S160A substitution. Of the six variable sites lining the chromophore‐binding pocket in SWS1, only one included a polarity change where species varied between S, T, and A at site 120.

### 3.4 Selective constraint and site‐specific positive selection

Overall, we found similar levels of average selective constraint among the four anuran visual opsins genes with SWS2 under the highest constraint (M0 *ω* = 0.089), RH1 under the lowest (M0 *ω* = 0.10375), and LWS and SWS1 intermediate (M0 *ω* = 0.097 and 0.010, respectively; Schott et al., 2022a). Results from using either the evolutionary topology or gene tree topologies were very similar and do not change the interpretations of the results, indicating that the results are robust to minor differences in topology (Schott et al., 2022a). Using the PAML M8 model we found statistically significant positive selection at a small proportion of sites in both anuran RH1 and LWS with both the ML gene tree and evolutionary tree topologies (Tables 4 and 5, Schott et al., 2022a). Four RH1 sites were inferred to be under positive selection with a BEB posterior probability of >80% (39, 107, 213, 270; Table 6). None of those sites have previously been identified to affect spectral tuning, but most are near known sites. FUBAR analysis identified one of the same sites as M8 (213) in addition to five other sites (65, 97, 112, 169, 277; Table 6, Schott et al., 2022a). BEB analyses of the PAML M8 model inferred two LWS sites to be under positive selection with posterior probability >80% (49, 217), while FUBAR identified three (49, 154, 166; Table 6). One of these (49) is a known spectral tuning site in the RH2 and SWS1 opsins. No evidence of positive selection in SWS1 or SWS2 was detected with the PAML models, although two sites were identified with greater than 90% posterior probability in SWS1 and one site in SWS2 using FUBAR (Table 6, Schott et al., 2022a).

**TABLE 4:** Results of PAML analyses performed on RH1 using the species topology. Results using the RH1 ML gene tree are similar and can be found on Zenodo (Schott et al., 2022a). Bold values indicate significant *p*‐values at the .05 significance level

Model	np	lnL	*k*	Parameters				Null	LRT	*df*	*p*
M0	61	−6926.03	2.06	0.10375				n/a			
M1a	62	−6700.83	2.12	p:	0.874	0.126		M0	450.396	1	.**0000**
				w:	0.031	1.000					
											
M2a	64	−6700.83	2.12	p:	0.874	0.002	0.124	M1a	0.000	2	1.0000
				w:	0.031	1.000	1.000				
											
M2a_rel	64	−6670.57	2.05	p:	0.700	0.070	0.230	M1a	60.523	2	.**0000**
				w:	0.002	1.000	0.204				
											
M3	65	−6668.94	2.02	p:	0.655	0.244	0.101	M0	514.188	4	.**0000**
				w:	0.000	0.139	0.774				
											
M7	62	−6671.09	2.02	p:	0.10616	q:	0.75681	n/a			
											
M8a	63	−6667.22	2.01	p:	0.133	q:	1.585	n/a			
				p1:	0.040	w:	1.000				
											
M8	64	−6664.44	2.03	p:	0.122	q:	1.156	M7	13.298	2	.**0013**
				p1:	0.014	w:	1.827	M8a	5.566	1	.**0183**

**TABLE 5:** Results of PAML analyses performed on LWS using the species topology. Results using the LWS ML gene tree are similar and can be found on Zenodo (Schott et al., 2022a). Bold values indicate significant *p*‐values at the .05 significance level

Model	np	lnL	*k*	Parameters				Null	LRT	*df*	*p*
M0	55	−7462.48	2.13	0.09662				n/a			
M1a	56	−7280.33	2.23	p:	0.867	0.133		M0	364.292	1	.**0000**
				w:	0.033	1.000					
											
M2a	58	−7278.72	2.24	p:	0.866	0.131	0.003	M1a	3.227	2	.1992
				w:	0.034	1.000	3.590				
											
M2a_rel	58	−7228.79	2.08	p:	0.203	0.031	0.766	M1a	103.075	2	.**0000**
				w:	0.320	1.000	0.012				
											
M3	59	−7228.61	2.08	p:	0.770	0.206	0.024	M0	467.743	4	.**0000**
				w:	0.013	0.338	1.159				
											
M7	56	−7232.66	2.08	p:	0.14811	q:	1.13691	n/a			
											
M8a	57	−7229.25	2.08	p:	0.174	q:	1.866	n/a			
				p1:	0.024	w:	1.000				
											
M8	58	−7227.31	2.09	p:	0.159	q:	1.373	M7	10.712	2	.**0047**
				p1:	0.004	w:	2.476	M8a	3.878	1	.**0489**

**TABLE 6:** Opsin amino acid sites inferred to be under positive selection with at least 80% posterior probability by either the BEB analyses of M8 model or with FUBAR. Sites numbers are relative to those in bovine RH1. Full PAML and FUBAR results tables can be found on Zenodo (Schott et al., 2022a)

Opsin	Site number	PAML M8 BEB		FUBAR	
		Posterior probability	*ω*	Posterior probability	*ω*
RH1	39	0.988	1.498 ± 0.106	0.791	3.272
RH1	65	–	–	0.865	3.005
RH1	97	–	–	0.862	2.777
RH1	107	0.979	1.192 ± 0.124	0.019	0.399
RH1	112	–	–	0.92	4.158
RH1	169	0.753	1.314 ± 0.339	0.987	8.027
RH1	213	0.964	1.482 ± 0.145	0.94	5.555
RH1	270	0.842	1.387 ± 0.283	0.15	0.878
RH1	277	–	–	0.885	3.475
LWS	49	0.995	1.524 ± 0.238	0.917	6.790
LWS	154	0.551	1.153 ± 0.401	0.918	4.358
LWS	166	–	–	0.864	2.830
LWS	217	0.898	1.442 ± 0.254	0.543	2.240
LWS	49	–	–	0.917	6.790
LWS	154	–	–	0.918	4.358
LWS	166	0.864	2.830258	0.864	2.830
SWS1	120	–	–	0.909	7.280
SWS1	159	–	–	0.906	3.560
SWS2	−2	–	–	0.901	7.194

## 4 DISCUSSION

Using a combination of retinal cDNA sequencing and previously published genomic and transcriptome resources, we obtained visual opsin genes for 33 anuran species spanning 12 families. We found that anurans generally possess four of the visual opsins common to vertebrates (*RH1*, *LWS*, *SWS1*, *SWS2*) with no evidence of the *RH2* opsin gene. While we had variable recovery of opsins from retinal cDNA, we did not find any evidence for loss of visual opsins in any of the species for which genomic data were available. We identified a single gene duplication, in *Pyxicephalus adspersus*, where a distinct *LWS* gene was found on each of the two sex chromosomes (Z and W). Overall, we found considerable variation in each of the four opsins across anurans at both previously known and potentially newly identified functional sites. In addition, we found evidence for positive selection in *RH1* and *LWS* at a small subset of sites. Below we discuss these findings in terms of how they may affect spectral tuning and dim‐light adaptation in anurans that inhabit different light environments.

### 4.1 Spectral tuning variation in anuran visual opsins

We identified considerable variation in each of the four visual opsins at known spectral tuning sites. However, much of this variation was between residues not found, or at least not explored, in other vertebrate groups making it difficult to predict the effect of the differences in protein sequence in anurans. In addition, the relative lack of data on visual pigment spectral absorbances available for anurans further limits our ability to infer the effect of particular substitutions on the spectral absorbance of the visual pigment. For each opsin, we also found variation at spectral tuning sites that are known from other visual opsins. While some of these sites are likely to affect spectral tuning in multiple visual opsins, others will have a more restricted effect due to interactions with other residues in the protein. Thus, our results highlight that there is likely considerable unappreciated variation in the spectral absorbances of anuran visual pigments, and we have identified numerous candidates for further functional studies.

Based on the limited available data, the RH1 visual pigment of most frogs, including *Lithobates* spp., *Bufo* spp., and *Hyla cinerea*, are reported to have a *λ*_max_ of ~502 nm. Exceptions to this are *Oophaga pumilio* with a *λ*_max_ of 491 nm and *X*. *laevis* with a *λ*_max_ of 535 nm (Siddiqi et al., 2004; Witkovsky et al., 1981). Unfortunately, we did not have an *O*. *pumilio* sample (or other dendrobatid) to evaluate potential causes of the blue‐shifted *λ*_max_. In *X*. *laevis*, the red‐shifted *λ*_max_ is caused by the use of a different chromophore that is derived from vitamin A_2_ (3,4‐didehydroretinal, referred to as A_2_), as opposed to the more typical A_1_ chromophore (retinal) used by most vertebrates (Bridges, 1972). The A_2_ chromophore is found in some anuran tadpoles but is replaced by A_1_ chromophore in the adults of most frog species, whereas other frogs exclusively use A_1_ in both larval and adult stages (Bridges, 1972). In *X*. *laevis*, however, A_2_ is used throughout its lifecycle (Bridges et al., 1977), which results in a *λ*_max_ of 524 nm (Witkovsky et al., 1981). Near complete replacement of A_2_ by A_1_ in *X*. *laevis* resulted in a *λ*_max_ of 503 nm for the RH1 visual pigment (Witkovsky et al., 1978) suggesting that the *X*. *laevis* RH1 opsin has similar spectral tuning to most other known frog RH1s. This is supported by our analysis where we found that *X*. *laevis* RH1 did not differ in any known RH1 tuning sites from the other species in our dataset with measured *λ*_max_ (e.g., *Lithobates* spp.). However, we did find that *X*. *laevis* differed from *Lithobates* spp. at five of the nine sites identified as being positively selected in RH1 (sites 39, 107, 112, 169, 213) suggesting that these sites may influence other aspects of visual pigment function. In particular, Q107P and L213T may be of particular interest for future studies.

The LWS cones of anurans, again based on limited data, have variable spectral sensitivities ranging from *λ*_max_ of 561–579 nm for A_1_‐based pigments. Unfortunately, sequences for *O*. *pumilio* and *R*. *temporaria*, which are reported to have blue‐shifted *λ*_max_ around ~561 nm (Koskelainen et al., 1994; Siddiqi et al., 2004) are not available, but the LWS‐specific spectral tuning substitution S164A likely contributes to this shift. Species with *λ*_max_ ≥ 570 nm (e.g., *Lithobates catesbeianus* and *L*. *sphenocephalus*; Hárosi, 1982; Liebman & Entine, 1968; Schott et al., 2021) have S164, and the substitution S164A was shown to shift *λ*_max_ −7 nm when mutated in human LWS (Asenjo et al., 1994). However, this substitution alone is not enough to account for the known variation in sensitivity, and thus substitutions at other sites are likely to also affect LWS spectral tuning in anurans. The four sites identified in anuran LWS as being positively selected are likely also to play a role, especially site 49, which was highly variable and is known to effect spectral tuning in other visual opsins.

Evidence for SWS1 cones in anurans was previously very limited (Hisatomi et al., 1998; Starace & Knox, 1998). While our data cannot inform on potential combinations of visual pigments in different types of cones, the fact that *SWS1* does not appear to have been lost in any species, and is under similar selective constraint as the other visual opsins, suggests that SWS1 visual pigment is present in anuran photoreceptors, at least at some point in their life cycle. This further suggests that SWS1 cones are common among anurans and are just difficult to detect with methods such as microspectrophotometry (MSP) and electroretinograms (ERG). A potential convergence of the *λ*_max_ of SWS1 and SWS2 (see above) may further complicate this, although in *X*. *laevis* the *λ*_max_ of these pigments expressed *in vitro* differed by 9 nm (425 vs. 434 nm, respectively; Darden et al., 2003; Starace & Knox, 1998). It is also possible that SWS1 is co‐expressed with another opsin as is the case in the cones of salamanders and several other vertebrates (Dalton et al., 2014; Isayama et al., 2014). Another possibility is that SWS1 is only expressed at certain life stages, for instance in tadpoles. Ontogenetic shifts in expression of visual opsins are fairly common in teleost fishes (Carleton et al., 2020), but the only study of expression profiles in a frog (*L*. *sphenocephalus*) found that *SWS1* was expressed at a low, but consistent level in both tadpoles and post‐metamorphic juvenile frogs (Schott et al., 2021).

Overall, the current literature suggests anuran SWS1 *λ*_max_ is fairly conserved and varies only between 425 and 433 nm, and yet our molecular data showed that SWS1 was the most variable of the four visual opsins at known spectral tuning sites. While this high sequence diversity perhaps indicates more variation in *λ*_max_ than is currently documented, we found that anuran *SWS1* was under high selective constraint and had little evidence of positively selected sites. Thus, potential spectral shifts may have only occurred a small number of times, in specific lineages, which would not leave a signature of positive diversifying selection detectable by the codon models we employed. Estimating the effect on spectral tuning of variation at known spectral tuning sites remains challenging because many of the sites have interacting effects, and, in some cases, the specific residues found in anurans are not found in other groups (Hauser et al., 2014; Takahashi & Yokoyama, 2005). Finally, studies of SWS1 *λ*_max_ in anurans to date have not yet found evidence that spectral sensitivity of this visual opsin is shifted into the ultraviolet. Shifts between violet and ultraviolet sensitivity are relatively common in vertebrates, especially in birds where studies support at least 14 shifts between violet and ultraviolet sensitivity (Ödeen & Håstad, 2013). While several of the changes we identified suggest ultraviolet sensitivity of SWS1 in anurans may be possible, further functional studies will be required to answer this question.

Uniquely in anurans and some salamanders the SWS2 opsin is expressed in SWS2 rods (also known as “green” rods; Hisatomi et al., 1999; Ma et al., 2001). In salamanders, the SWS2 opsin is also expressed in cones, but direct evidence of SWS2 cones is lacking in anurans (Darden et al., 2003; Hisatomi et al., 1999; Isayama et al., 2014; but see Siddiqi et al., 2004). The *λ*_max_ of anuran SWS2 rods, at least based on current data, is conserved around ~432 nm (e.g., Govardavskii et al., 2000; Hárosi, 1982; Liebman & Entine, 1968). *Lithobates catesbeianus* and *R*. *temporaria* also have cones with the same *λ*_max_ as the SWS2 rods, although immunohistochemical evidence in *L*. *catesbeianus* shows no evidence of SWS2 expression in cones, suggesting that the SWS1 and SWS2 pigments may have converged on the same *λ*_max_ (Donner & Yovanovich, 2020; Hárosi, 1982; Koskelainen et al., 1994). The *λ*_max_ of SWS2 rods in *X*. *laevis* was estimated to be 445 nm with A_2_ (Witkovsky et al., 1981), but when the SWS2 pigment was expressed *in vitro* with the A_1_ chromophore the *λ*_max_ (434 nm) that of other anuran species that use the A_1_ chromophore. *Xenopus laevis* and the other species for which SWS2 *λ*_max_ has been estimated (e.g., *L*. *catesbeianus*, *Bufo bufo*; Govardovskii et al., 2000; Hárosi, 1982) differ at the SWS2 spectral tuning site 122 (I in *X*. *laevis*, M in the others). In the newt *Cynops pyrrhogaster* I122M resulted in a −6 nm shift (Takahashi & Ebrey, 2003), which suggests that the spectral tuning effect of this site may differ between anurans and salamanders. *Xenopus laevis* and the other species also differed at a number of spectral tuning sites known from other opsins, but given the similar values of *λ*_max_ among species, these sites are unlikely to affect spectral tuning in anuran SWS2. The absorbance spectra of *O*. *pumilio* cones, however, do hint at the potential for substantial variation in anuran SWS2. This species, which was found to lack “green” SWS2 rods, has cones with a *λ*_max_ of 466 nm that may contain a red‐shifted SWS2 pigment. Further studies are needed to explore the molecular mechanisms of this potential red‐shift and other spectral tuning mechanisms in anuran SWS2 pigments.

### 4.2 First evidence of visual opsin duplication in amphibians

We found the first evidence of a visual opsin gene duplication in amphibians in the African bullfrog, *Pyxicephalus adspersus*, with two copies of *LWS*, one on each of the sex chromosomes. Visual opsin gene duplication is rare among tetrapods having previously only been reported in some marsupials where *RH1* was duplicated (Cowing et al., 2008) and in two primate lineages where *LWS* was duplicated (Carvalho et al., 2017) but is common in teleost fishes (Carleton et al., 2020). The location of the *LWS* duplicates on different sex chromosomes in *P*. *adspersus* differs from the primate duplications where the two duplicates are found on the same sex chromosome (X) but could be functionally similar to the allelic variation in some primate LWS. In those primates, heterozygotes have two distinct *LWS* alleles on the X chromosomes that enable red‐green color discrimination in females, but not males (Carvalho et al., 2017). In *P*. *adspersus* the two *LWS* genes are on the Z and W chromosomes, respectively. Thus, males would have two copies of the same (Z) gene, while females would have two different copies (ZW) potentially enabling additional color discrimination if the *λ*_max_ of the two genes has diverged. The Z and W *LWS* genes have several nonconservative changes at the 17 sites where they differ, but these are not at any known spectral tuning or positively selected sites. Thus, the potential impact of these changes on tuning or other functional properties will require further study. A potential sex‐specific selective advantage of two *LWS* genes is also unclear but could be related to a behavior of females who will swim underwater to avoid smaller males in order to reach and mate with the larger, dominant male (AmphibiaWeb 2022). A second, red‐shifted LWS pigment could provide a visual advantage in the red‐shifted freshwater environments, something that is achieved through the use of the A_2_, instead of the A_1_, chromophore in the tadpoles of many species, and in a fully aquatic species such as *X*. *laevis*. *Pyxicephalus adspersus* is also one of a small number of diurnal frog species, which generally require further study to evaluate other potential adaptations to bright‐light and color vision in anurans.

### 4.3 Anuran visual opsins are under moderate selective constraint relative to other vertebrate groups

Previous studies have investigated selective constraint acting on visual opsins in other vertebrate groups such as mammals, reptiles (including birds), and teleost fishes, but no other studies have done so in anurans. Compared to these other groups we found that anuran visual opsin genes had moderate levels of selective constraint in line with those found more broadly across protein‐coding genes (*ω* from 0.08 to 0.18; Fay & Wu, 2003). We found that anuran *RH1* was under higher constraint (*ω* = 0.10) than in groups with high levels of positive selection such as cichlids (*ω* = 0.25–0.44; Hauser et al., 2017; Schott et al., 2014) and snakes (*ω* = 0.22; Schott et al., 2018), but under lower constraint than mammal *RH1* (*ω* = 0.04; Gutierrez, Castiglione, et al., 2018). Instead, selective constraint on anuran *RH1* was similar to that in reptiles (including lizards, snakes, turtles, crocodilians, and birds; *ω* = 0.11; Schott et al., 2018) and ray‐finned fishes (*ω* = 0.07–0.09; Rennison et al., 2012). Selective constraint in cone opsins has been less extensively studied, but anuran *LWS* (*ω* = 0.10) was under similar constraint to reptiles when snakes, which had high levels of positive selection, were excluded (*ω* = 0.08; Schott et al., 2019) and to bats (*ω* = 0.08; Gutierrez, Schott, et al., 2018). For *SWS1*, bats and anurans also showed similar levels of selective constraint (*ω* = 0.08 and 0.01, respectively; Gutierrez, Schott, et al., 2018), whereas *SWS1* was slightly more constrained in reptiles (*ω* = 0.06), especially with snakes removed (*ω* = 0.03; Schott et al., 2019). Selective constraint in anuran *SWS2* (*ω* = 0.09) was similar to that found both across reptiles (*ω* = 0.08; Gemmell et al., 2020) and specifically in warblers (*ω* = 0.05–0.07; Bloch et al., 2015). Comparatively, neotropical cichlids had less constrained *SWS1* (*ω* = 0.20) and *SWS2* (*ω* = 0.25–0.30) both of which were also found to be positively selected (Hauser et al., 2021).

In terms of positive selection, anurans showed both less pervasive positive selection (i.e., at a smaller proportion of sites) and weaker positive selection (lower *ω*) than other groups with visual opsins under strong positive selection. For example, neotropical cichlid *RH1* showed positive selection at 4% of sites with an *ω* of 5.4 (M8 model; Hauser et al., 2017) compared to only 1.4% of sites with an *ω* of 1.8 in anurans. Similarly, in snake LWS 10.9% of sites were found to be positively selected with an *ω* of 2.6 compared to 0.4% of sites in anurans, albeit with a similar *ω* of 2.5 (M8 model; Schott et al., 2018). Comparisons between anurans, snakes, and cichlids are not equal in terms of evolutionary scale, and thus it is difficult to draw meaningful conclusions from the differences we observed. Further sampling across anurans may reveal specific clades that are under strong positive selection and that are driving the overall signal of positive selection in anuran *RH1* and *LWS*.

### 4.4 Potential functional adaptations for dim‐light vision in anuran RH1 and SWS2

Most anurans are nocturnal, at least as adults, and thus we might expect their visual systems to be particularly adapted to vision in dim‐light conditions, and at the morphological and cellular levels, this appears to be the case. Many anurans have relatively large eyes (Thomas et al., 2020) as well as very large and numerous rod photoreceptors (Nilsson, 1964). Additionally, most anurans have a second type of rod photoreceptor (SWS2 rods), which may further enhance visual sensitivity and enable color discrimination at light levels where for most other animals only achromatic vision is possible (Yovanovich et al., 2017). We also identified several features at the molecular level that also may provide dim‐light adaptation. RH1 N83 has been identified as a dim‐light adaptation based on an accelerated formation of the active signaling state of the visual pigment (Sugawara et al., 2010). Mutations to N83 have also been shown to increase the time it takes for the chromophore to exit the binding pocket after light activation (retinal release rate), which could prolong the lifetime of the active state increasing light sensitivity (Bickelmann et al., 2012). All anurans in our sample had N83, including the two diurnal species in our dataset (*Pyxicephalus adspersus* and *Mantella baroni*), which could indicate this site has become fixed in frogs regardless of light environment. However, there is some disconnect between N83 and dim‐light environments because diurnal turtles and lizards have N83, while nocturnal crocodilians have D83 (Schott et al., 2018; Ryan K Schott personal observation), which may indicate that there are more complex functional roles of substitutions at this site that require further study. A second site, 299, was also shown to affect retinal release rate where the substitutions S299A and A299S increased and decreased retinal release rates, respectively (Dungan & Chang, 2017). Variation between S and A at site 299 also occurred in our sample of anurans, although interestingly S299 was not found in either of the diurnal species or those with partial daytime activity (e.g., *Lithobates* spp.). Thus, species with the combination N83 and S299, which when mutated in bovine RH1 resulted in the slowest retinal release rate (Dungan & Chang, 2017), were only found in nocturnal species and in particular included subfossorial and burrowing species (*Microhyla fissipes* and *Spea* and *Scaphiopus* spp.). Whether this is related to visual performance in these dim‐light habitats remains to be tested.

We also found that all anurans in our sample had SWS2 with T47 regardless of activity pattern. This residue was shown to result in increased light sensitivity through increased dark state stability (i.e., low thermal isomerization rate) to levels nearly as high as RH1 (Kojima et al., 2017). Extremely high dark state stability of RH1 is one of the functional properties that enable single photon responses in rods (Lamb, 2013), and thus is likely crucial for the function of SWS2 rods in dim‐light vision and would be necessary to achieve color vision at scotopic light levels (Yovanovich et al., 2017). While it has not been explored, this increased sensitivity likely comes with a trade‐off that reduces response times and/or recovery rates, which are much higher in cones (Lamb, 2013). It is unknown whether all the anurans in our sample have SWS2 rods, but data from *X*. *laevis* and *L*. *catesbeianus* suggest that SWS2 is present only in rods and not cones (Darden et al., 2003; Hisatomi et al., 1998, 1999; Starace & Knox, 1998). The only anuran species where SWS2 rods have been shown to be absent (*O*. *pumilio*; Siddiqi et al., 2004) lacks molecular data to determine whether SWS2 was lost or may instead be expressed in a cone. Interestingly, salamanders, which lack the T47 substitution, can have both SWS2 cones and SWS2 rods (Isayama et al., 2014; Ma et al., 2001). This suggests that SWS2 may be constrained in salamanders to function in both rods and cones, but that SWS2 has more completely adapted to a dim‐light, rod function in frogs. Further studies will be needed to explore whether there is indeed a functional trade‐off and if anuran species lacking SWS2 rods have undergone a reversal at site 47.

## 5 CONCLUSIONS

Anurans form a largely understudied but intriguing group of organisms for studies of visual system evolution, in part due to their reliance on visual cues and specialization for dim‐light vision, including the unique use of two spectrally distinct rod classes. Additionally, while most molecular vision studies have focused on organisms living in either aquatic or terrestrial light environments, anurans provide an opportunity to study species that ontogenetically transition between these very different light environments. Here we have performed the first analysis of visual opsin sequence diversity across anurans and found variation in both known and potential spectral tuning sites, as well as evidence for positive selection in *RH1* and *LWS*. This suggests substantial variation in spectral tuning among anurans, but the exact spectral tuning changes (or other functional changes) are difficult to predict. This is because most of the variants that occur at known spectral tuning sites in anurans are unique or have known affects only when combined with other specific residues. However, our results do suggest potential dim‐light functional adaptation in anuran RH1 and SWS2. We also found support for a functional and selectively constrained SWS1 visual pigment across anurans and the first evidence of opsin duplication in amphibians with the duplication of *LWS* on different sex chromophores in *Pyxicephalus adspersus* suggesting the possibility of sex‐specific visual adaptation in this species. Overall, our study provides a foundation to support future research into anuran visual ecology and evolution.

## CONFLICT OF INTEREST

The authors declare no conflict of interest.

## AUTHOR CONTRIBUTION

**Ryan K. Schott:** Data curation (lead); Formal analysis (lead); Investigation (equal); Validation (lead); Visualization (lead); Writing – original draft (lead); Writing – review & editing (lead). **Leah Perez:** Data curation (supporting); Formal analysis (supporting); Investigation (equal); Resources (supporting); Writing – original draft (supporting); Writing – review & editing (supporting). **Matthew A. Kwiatkowski:** Conceptualization (equal); Funding acquisition (equal); Project administration (supporting); Resources (supporting); Supervision (supporting); Writing – review & editing (supporting). **Vance Imhoff:** Data curation (supporting); Investigation (supporting); Validation (supporting); Writing – review & editing (supporting). **Jennifer M. Gumm:** Conceptualization (equal); Data curation (supporting); Funding acquisition (equal); Project administration (lead); Resources (lead); Supervision (lead); Validation (supporting); Writing – review & editing (supporting).

### OPEN RESEARCH BADGES

This article has earned an Open Data Badge for making publicly available the digitally‐shareable data necessary to reproduce the reported results. The data is available at https://doi.org/10.5281/zenodo.5252929.

## Data Availability

Sequences generated with this study have been deposited to NCBI Genbank under accession numbers OM243040–OM243087. Lab protocols used in the study have been deposited at protocols.io at: http://doi.org/10.17504/protocols.io.b3yqqpvw. All other data associated with the study, including full sequence data and results tables, multiple sequence alignments, phylogenetic tree topologies, and sequencing primers are available on Zenodo at http://doi.org/10.5281/zenodo.5252929.
